# A novel ligand-modified nanocomposite microparticles improved efficiency of quercetin and paclitaxel delivery in the non-small cell lung cancer

**DOI:** 10.1080/10717544.2022.2120567

**Published:** 2022-09-23

**Authors:** Xiaoming Cui, Fang Zhang, Yanyan Zhao, Pan Li, Ting Wang, Zhilu Xu, Jingjing Zhang, Weifen Zhang

**Affiliations:** a College of Pharmacy, Weifang Medical University, Weifang, P.R. China; b College of Basic Medical, Qingdao Binhai University, Qingdao, P.R. China; c Shandong Intelligent Materials and Regenerative Medicine Engineering Technology Research Center, Weifang, P.R. China

**Keywords:** NSCLC, nanocomposite microspheres, anti-tumor, active targeting, orthotopic

## Abstract

Chemotherapy is the first choice for the treatment of cancer but it is still limited by insufficient kill efficiency and drug resistance. These problems urgently need to be overcome in a way that minimizes damage to the body. In this study, we designed the nanocomposite microparticles (NMPs) modified by cetuximab (Cet) and loaded anti-tumor agents- quercetin (QUE) and paclitaxel (PTX)- for eliciting specific drugs homing and enhancing the killing efficiency of chemotherapy drugs (P/Q@CNMPs). Physicochemical characteristics results presented that P/Q@CNMPs have a suitable aerodynamic diameter and uniform morphology that could meet the requirements of particles deposition in the lung. And it also had the characteristics of sustained-release and pH-responsive which could release the agents in the right place and has a continuous effect. *In vitro* and *in vivo* analysis results presented that P/Q@CNMPs have the accuracy targeting ability and killing effect on non-small cell lung cancer (NSCLC) which express positive epidermal growth factor receptor (EGFR) on the membrane. Furthermore, this system also has low toxicity and good biocompatibility. These results demonstrated that P/Q@CNMPs could be a potential intelligent targeting strategy used for chemo-resistant NSCLC therapies.

## 1. Introduction

The standard treatment methods for non-small cell lung cancer (NSCLC) include surgery, radiotherapy, and chemotherapy of which chemotherapy is the most drastic step (Roy & Li, 2016). However, the application of chemotherapy was limited by the insufficient drug concentration in tumor cells, unnecessary drug damage to normal cells, and drug resistance (Goldman et al., 2015).

Nanomedicines have been intensively investigated as new therapeutic options for cancer treatment (Zhen et al., 2013; Araste et al., 2021; Hidalgo et al., 2021), such as organic nanoparticles (NPs), inorganic nanoparticles, micelles, liposomes, ect (Yan et al., 2019; Li et al., 2021; Liang et al., 2021), but the low specific targeting efficiency problem and toxicity still existed. Therefore, the ligand modified on the surface of NPs has an important function, driving the delivery system to track tumor cells, and has been developed to overcome the various limitations of conventional chemotherapeutics (Yang et al., 2015; Yoo et al., 2019; Hu et al., 2021). In this account, we designed the nanocomposite microparticles co-loaded quercetin (QUE) and paclitaxel (PTX) and functioned with cetuximab (P/Q@CNMPs) to enhance the active targeted and killing effect to tumor cells. This delivery system can be administered by inhalation to the tumors in the lungs which can accurately reach the lesion site.

Epidermal growth factor receptor (EGFR) overexpression in the NSCLC has been proved in some research (Kwok & Chan, 2013; Zhou et al., 2020). Cetuximab (Cet) is a recombinant human/mouse chimeric EGFR monoclonal antibody that has a good clinic effect on the early stage of metastatic colorectal cancer and head and neck cancer (Vermorken et al., 2008). Cet has also been combined with chemotherapeutic drugs as the first choice for the treatment of lung cancer (Sreeranganathan et al., 2017; Zhang et al., 2019).

PTX, which is used to treat various types of cancer, is also a first-line chemotherapy agent for NSCLC. It has been shown to interfere with the normal function of microtubules during cancer cell division (Yahya et al., 2019). Two major factors limited the application of PTX (Ryu et al., 2012; Agarwal et al., 2018). The first one is PTX’s damage effect also present on normal rapidly dividing cells such as multipotential stem cells, and the other factor is the acquired resistance which is mainly ascribed to the increased efflux of P-gp protein triggered by the self-protection mechanism of tumor cells. These side effects will lead to lower effects and more toxicity. Therefore, measures for targeting the delivery of PTX to the tumor and keeping the sensibility of cancer cells to PTX urgently need to be developed.

Studies have shown that QUE works by interfering with the hydrolysis activity of ATPase in a similar way as noncompetitive binding and blocking the efflux function of P-gp which could alleviate the drug resistance of PTX (Yu et al., 2019). Furthermore, the pulmonary drug delivery systems (PDDs) can deliver drugs directly to the lung lesions, reducing the toxic effect of chemotherapy drugs on other normal tissues and increasing the treatment accuracy (Zhang et al., 2021). The particles with diameters of 1–5 µm were suitable for aerosols intended for inhalation while the nanomedicines would be easily exhaled from the lungs with airflow. Particle size greatly affect the tumor penetration of nanoparticles and small size particles has more penetration (Hu et al., 2021), paradoxically, micro-size medicines have lower uptake efficiency by cancer compared with nanoparticles (NPs). Therefore, nanoparticle-composite microspheres (MNPs) were developed which can disperse from MPs to NPs and could combine the advantages of nano- and micro-size particles (Liang et al., 2019). And the characteristic of MNPs quick disperse from MPs into NPs was due to the selected excipients - mannitol with strong water absorption and easy disintegration, can be ensured the MNPs rapidly decomposition under moisture environment of mucus and pulmonary surfactant in the lungs that lead to the drug-loaded NPs release from MPs system (Wang et al., 2016; Sun et al., 2019).

In this study, we designed the Cet modification on the PTX/QUE-loaded nano-composite microparticles (P/Q@NMPs) to obtain P/Q@CNMPs which is an active targeted delivery system, playing a role in the NSCLC of EGFR high expression. This drug delivery system was expected to enhance the accuracy therapeutic effect of the positive expression of EGFR NSCLC cells, reduce the toxicity on other major organs, and improve the application of the low solubility chemotherapy drugs.

## 2. Materials and methods

### 2.1. Materials

Chitosan (96.1% degree of deacetylation, molecular weight 120, 000) was purchased from Hai Debei Marine Biotechnology Company (Jinan, China). PTX was obtained from Shanghai Yuanye Bio-Technology (Shanghai, China). QUE was purchased from Shanxi Langsen Biotechnology (Taiyuan, China). Sodium tripolyphosphate (TPP) was obtained from Kelong Chemical (Chengdu, China). Cetuximab was purchased from Merck (Germany). All other chemicals were of chromatographic grade.

### 2.2. Cell culture

Human NSCLC cell lines A549 (EGFR high expression) and H1299 (EGFR low expression) were acquired from American Type Culture Collection (Manassas, VA, USA). All cell lines were maintained in RPMI 1640 supplemented with 12% fetal bovine serum (FBS; Invitrogen, CA, USA), 1% sodium pyruvate, and 1% antibiotic/antimycotic solution and maintained at 37 °C with 5% CO_2_ incubator.

### 2.3. Synthesis of Cet-CTS and preparation of P/Q@CNMPs

Cetuximab modified chitosan (Cet-CTS) was synthesized in advance to prepare Cet-modified P/Q@CNMPs and the Michael addition method was used (Li et al., 2020). Firstly, a certain amount of sulfosuccinimidyl 4-[N-maleimidomethyl] cyclohexane-1-carboxylate (Sulfo-SMCC, 1 mg/mL) was added to the CTS solution (1 mg/mL). The amine-reactive N-hydroxysuccinimide (NHS ester) of Sulfo-SMCC reacted with the primary amine of CTS to form a stable amide bond. The reaction was stirred at room temperature for 30 min. After that, the mixture was dialyzed in deionized water with a dialysis bag (3,500 molecular weight cutoff, membrane-cell, Chicago, IL, United States) for 48 h and lyophilized.

PTX-loaded NPs (PNP) modified by Cet to obtain PTX-loaded Cet-NPs (PCNP) and QUE-loaded NPs modified by Cet to obtain QUE-loaded Cet-NPs (QCNP). The NPs were prepared according to the ionic crosslinking method. 1 g Cet-CTS was dissolved in 1% (w/v) acetic acid solution, and ammonia solution (1%, v/v) was used to adjust the solution pH to 5.5. PTX and QUE solution were mixed with the above Cet-CTS suspension and TPP solution (1 mg/mL) was added by drop, sheared for 2 min, and magnetically stirred for 30 min at room temperature, resulting in drug-loaded NPs suspensions.

And then, spray-drying technology was used to composite NPs to P/Q@CNMPs and P/Q@NMPs. The mass ratio of various matrices of mannitol (MNT), lactose (LTS), and hydroxypropyl-β-cyclodextrin (HP-β-CD) was 3:1:1 (Liu et al., 2017). FITC-labeled QCNP and TRITC-labeled PCNP have been used for imaging the distribution of these two NPs in P/Q@CNMPs. In the experiments, Cy5.5-labeled NMPs were used for *in vivo* imaging of the targeting function P/Q@CNMPs. All formulations were spray-dried at an inlet temperature of 130 °C, the outlet temperature of 70 °C, pumping power at 50%, feed speed at 5 mL/min, and air atomization flow rate at 301 L/h. After the spray drying, the prepared dried microparticles (MPs) were removed from the sample collector and stored in the sample collection bottle.

### 2.4. Characterization of P/Q@NMPs and P/Q@CNMPs

The particle size and Zeta potential of P/Q@NMPs and P/Q@CNMPs were determined through the dynamic light scattering (DLS) measurements with a Malvern Nano-ZS 90 instrument (Malvern Pana transmission electron microscopy lyrical, Westborough, MA, USA). The morphology was observed by transmission electron microscopy (TEM, JEM-1230; JEOL, Tokyo, Japan) and scanning electron microscopy (SEM, Hitachi High-Tech Science Corporation, Tokyo, Japan). Aerodynamic diameter (d_aer_) was also determined by placing 0.5 g samples in the pycnometer (5 mL) and dropped in the air at a frequency of 30 times/min (height of about 14 cm) until the powder volume in the pycnometer was no change be observed (*n* = 3). The drug loading (DL) and encapsulate efficiency (EE) were determined by Ultraviolet (UV). The DL, EE, and aerodynamic diameter (d_aer_) were calculated according to the following equations:

(1) DL(%)=DlWf×100%

(2) EE(%)=DlDt×100%

(3) $${\;d}_{\mathrm{aer}}=\mathrm{d}\sqrt{\frac{\rho }{\rho_{1}}}$$

where D*_l_* is the determined weight of the drug in MPs, W_f_ is the total weight of the drug-loaded MPs, and D_t_ is the total weight of the number of drugs added. d_aer_ is the aerodynamic diameter of MPs, d is the geometric particle size of MPs, *ρ* is the tapped density of MPs, and *ρ*_1_ is the water mass density (*ρ*_1_=1 g/cm^3^). Each experiment was repeated thrice independently.

### 2.5. *In vitro* release study

The *in vitro* drug release behavior analysis was performed using the dialysis method (Shahin et al., 2013) in PBS (pH 7.4, 6.8, 5.5). P/Q@CNMPs were placed in the dialysis bag with 2 mL PBS (pH 7.4, 6.8, 5.5) and dipped into 250 mL conical flasks containing 50 ml of the same pH release medium. And then incubated the conical flasks on an incubator at 100 rpm, 37 °C. The sample was taken 1 mL from the release medium at specific time points (0.5, 0.75, 1, 2, 3, 4, 6, 8, 10, 12, 24, 48 h) and immediately replaced with 1 mL fresh release medium. UV was used to detect the cumulative dose of PTX and QUE, respectively. Each experiment was repeated thrice independently.

### 2.6. Anti-tumor efficacy of P/Q@CNMPs in vitro

The live/dead cells were evaluated using Calcein AM/PI double staining. A549 cells were seeded into 24-well culture plates (5 × 10^4^ cells/per well) and incubated with different drug formulations for 48 h, including control, pure PTX, pure QUE, the mixture of PTX and QUE, P/Q@NMPs, and P/Q@CNMPs groups. The fluorescence intensity was evaluated with an inverted fluorescence microscope (IX71; Olympus, Tokyo, Japan). Each experiment was repeated thrice independently.

Cell colony assay was also present to further confirm the anti-tumor efficacy of P/Q@CNMPs. A549 cells were seeded into 6-well culture plates (6 × 10^2^ cells/per well and incubated with different drug formulations. After 6 h incubation, the medium with drugs was removed and replaced with a fresh medium. After 2 weeks, cells were treated with4% paraformaldehyde and 0.1% crystal violet (MedChemExpress). Then cell colonies were calculated and took pictures in every experiment group. Experiments were performed thrice independently.

### 2.7. Targeting effect of P/Q@CNMPs in vitro

To evaluate the targeting effect of P/Q@CNMPs on NSCLC, A549 and H1299 cell lines were selected and seeded on coverslips in 6-well plates (2 × 10^5^ cells/well) and co-cultured with P/Q@NMPs and P/Q@CNMPs for 1, 2, 4 h, respectively. Following, cells were fixed with PFA, 4% for 15 min and stained with Hoechst for 10 min at room temperature, for confocal fluorescence imaging.

### 2.8. Biodistribution and anti-tumor efficacy of P/Q@CNMPs in vivo

All animal experimental protocols were approved by the Ethics Committee for the Use of Experimental Animals of Weifang Medical University (2020SDL178). Thirty 6-week BALB/c nude mice were obtained from Beijing Vital River Laboratory Animal Co., LTD. The NSCLC orthotopic tumor-bear mice model was established by injecting 1.0 × 10^6^ A549-LUC cells (25 μL) mixed with matrigel in an equal volume (Corning, cat. no. 354234), and insulin injection needles were used to inject the mixture into the left lung. After 5 weeks, the tumor size in tumor-bearing mice was detected by IVIS Spectrum (Perkin Elmer Instrument Co. LTD, USA).

### 2.9. In vivo imaging in tumor-bearing mice

A biodistribution experiment was used to evaluate the targeting efficiency of the P/QNMPs and P/Q@CNMPs *in vivo*. A549-Luc tumor-bearing mice were randomly divided into three groups (*n* = 3): free Cy5.5 group, Cy5.5@NMPs group, and III: Cy5.5@CNMPs group. Cy5.5 is a lipid-soluble fluorescent probe designed to simulate the distribution of lipid-soluble PTX and QUE *in vivo*. The distribution of fluorescence inside tumor-bearing mice was detected by IVIS Spectrum (Perkin Elmer Instrument Co. LTD, USA) at 1, 2, 4, 8, 12, 24, and 48 h after administration. After 48 h of the observation, the mice of each group were sacrificed to test the distribution of Cy5.5-labeled MPs in tumor tissues and primary organs.

### 2.10. Anti-tumor efficacy in tumor-bearing mice

The tumor-bearing mice were randomly divided into 6 groups: control group, pure PTX group, pure QUE group, the mixture of PTX and QUE group, P/Q@NMPs group, and P/Q@CNMPs group (*n* = 5), respectively. Every group was treated every three days. The dosage of PTX was 10 μg/g and QUE was 80 μg/g. All mice were sacrificed on day 22, and blood and main organs were collected. The heart, liver, spleen, lung, kidney, and tumor tissue of tumor-bearing mice were performed for histopathological examination by Hematoxylin and eosin (H&E) staining.

### 2.11. Hematological analysis

The hematological analysis was assayed by the blood count analysis and the commercial enzyme-linked immunosorbent assay (ELISA) kit. The concentration of IL-12/p40, BUN, ALT, and Cr was assessed according to the manufacturer’s instructions. The blood samples were collected from treatment nude mice and were centrifuged at 12, 000 g for 10 min. The serum was extracted from the top layer and stored at 4 °C. We also process the blood cell assay by the DxH900 Hematology Analyzer (Beckman Co. LTD, USA).

### 2.12. Statistical analysis

All experiments were performed in triplicates, and the obtained values were expressed as mean ± standard deviation (SD). All data were analyzed using the Statistical Package for the Social Sciences (SPSS) 19.0 (SPSS Inc., Chicago, IL, United States); the Student’s *t-*test and one-way ANOVA were used to determine the statistical significance. A *p-*value of *<* 0.05 was considered statistically significant.

## 3. Results

### 3.1. Preparation and characterization of the P/Q@NMPs and P/Q@CNMPs

**Figure 1.**
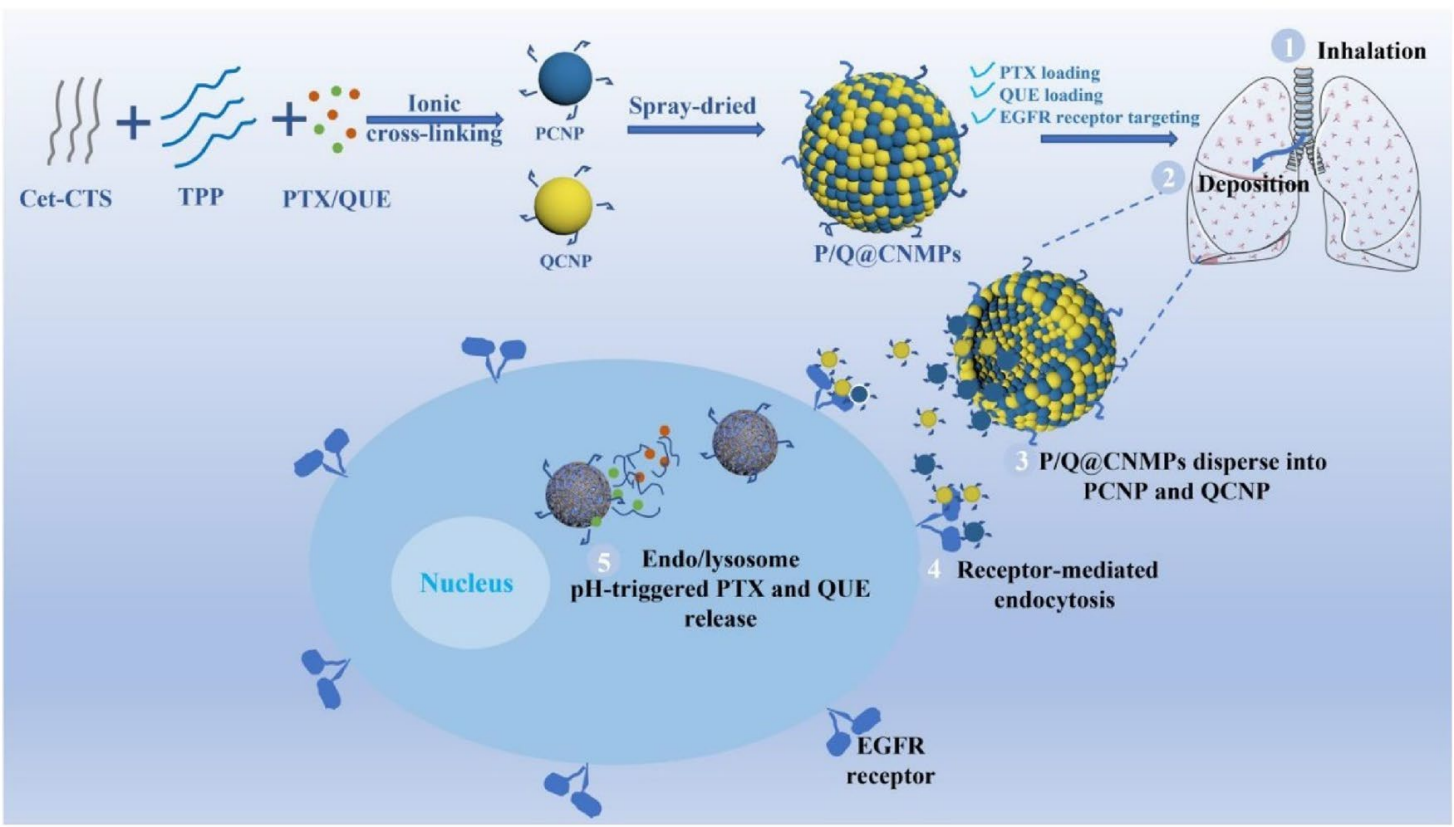
Schematic representation of the P/Q@CNMPs synthesis and targeting mechanism to tumor tissue.

The preparation route and the targeting mechanism for tumor cells of blank NMPs, P/Q@NMPs, and P/Q@CNMPs were shown in Figure 1. The mean hydrodynamic diameters of NMPs, P/Q@NMPs, and P/Q@CNMPs were 3.48 ± 0.55, 3.86 ± 0.29, and 4.54 ± 0.30 μm, respectively; and the corresponding value of aerodynamic diameter (d_aer_) was 2.09 ± 0.33, 2.12 ± 0.16 and 2.91 ± 1.94 μm, respectively (Table 1). The mean Zeta potential of all types of MPs was positive. The EE rate of PTX in P/Q@NMPs and P/Q@CNMPs was 80.94 ± 1.54% and 87.48 ± 7.10%, respectively. As for the EE rate of QUE, these values were 73.10 ± 1.78% and 86.47 ± 5.56. At the same time, the DL rate of PTX in P/Q@NMPs and P/Q@CNMPs was 3.45 ± 0.10% and 3.7 ± 0.30% while the QUE values were 3.1 ± 0.10% and 3.7 ± 0.23%, respectively (Table 1).

**Table 1.** The particle size, Zeta potential, and aerodynamic diameter of different MPs.

Sample	Size (μm)	Zeta (mV)	d_are_ (μm)	Drug loading (%)	Encapsulation efficiency (%)
P/Q@NMPs	3.86 ± 0.29	5.52 ± 0.45	2.12 ± 0.16	3.45 ± 0.1 (PTX) 3.1 ± 0.1 (QUE)	80.94 ± 1.54 (PTX) 73.1 ± 1.78 (QUE)
P/Q@CNMPs	4.54 ± 0.30	22.93 ± 1.48	2.91 ± 1.94	3.7 ± 0.3 (PTX) 3.7 ± 0.23 (QUE)	87.48 ± 7.1 (PTX) 86.47 ± 5.56 (QUE)

**Figure 2.**
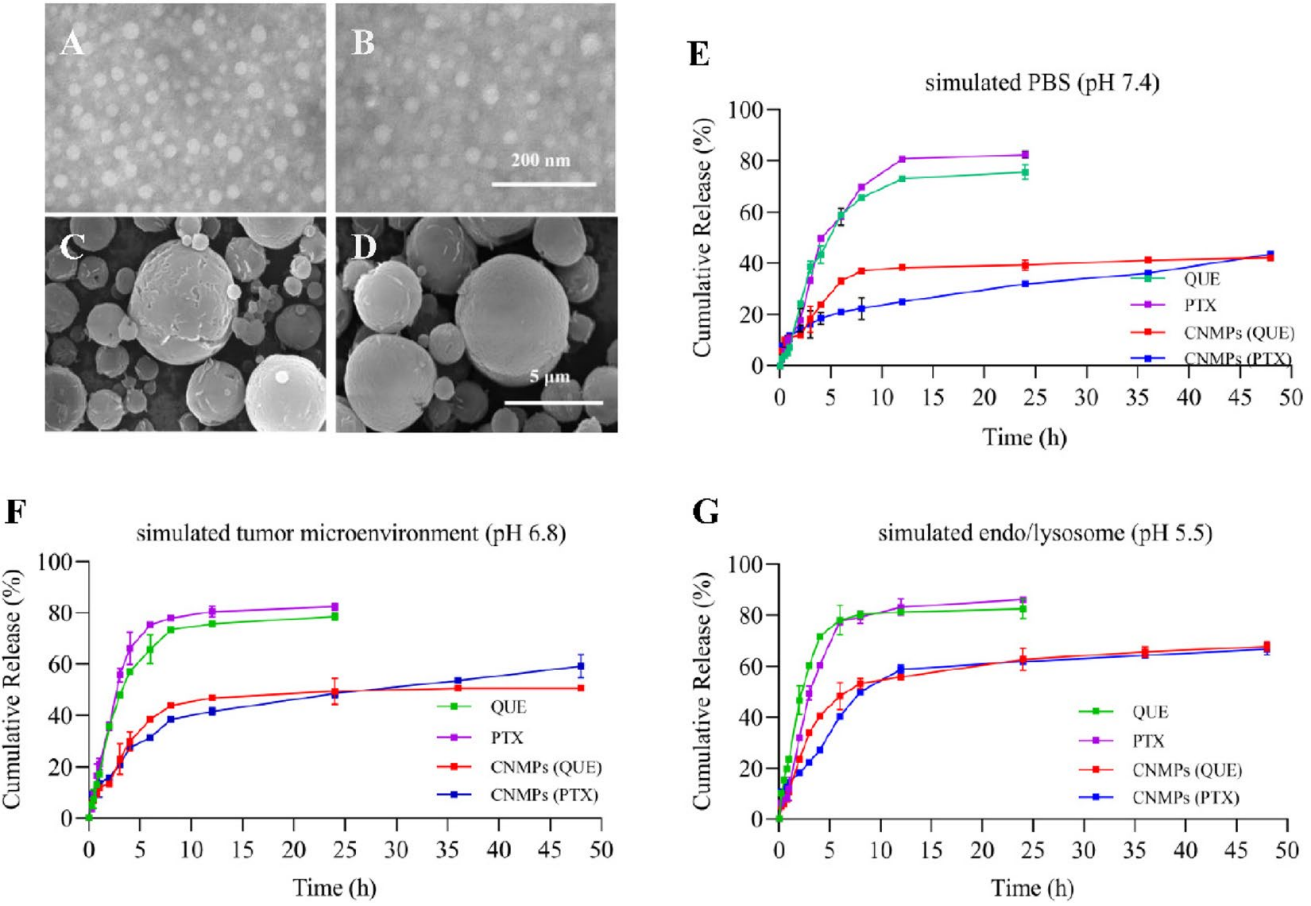
Characteristics of the P/Q@CNMPs. TEM microphotographs of the (A) PNP + QNP and (B) PCNP + QCNP, scale bar is 200 nm; SEM microphotographs of (C) P/Q@NMPs and (D) P/Q@CNMPs, scar bar is 5 μm; PTX and QUE release behavior in (E) PBS (pH 7.4), (F) simulated tumor microenvironment (pH 6.8), and (G) simulated endo/lysosome (pH 5.5) (*n* = 3).

Figure 2(A) and (B) gave the TEM microphotographs showed that PNP + QNP, and PCNP + QCNP had uniform size distribution and particle size around 30 nm. Figure 2(C) and (D) showed that SEM microphotographs of P/Q@NMPs, and P/Q@CNMPs had a spherical morphology, rough surface, and size mostly arranged from 1 to 5 μm which is consistent with the d_aer_ result.

### 3.2. In vitro release behavior studies

The release spectrum of PTX and QUE from P/Q@CNMPs and free drugs was determined in pH 7.4 (PBS), pH 6.8 (simulated tumor microenvironment), pH 5.5 (simulated endo/lysosome). Free PTX and QUE were almost totally released in the first 10 h. In the contrast, the PTX and QUE release from P/Q@CNMPs was sperate in two stages, burst and sustained release. In Figure 2(E)–(G), firstly, the PTX released from P/Q@CNMPs reached to 14.15%, 15.75%, and 18.22% while the QUE release reached 11.93%, 22.39%, and 23.58% in PBS of pH 5.5, pH 6.8, and pH 7.4 at 2 h, respectively. This result presented a burst release stage. And then, PTX and QUE release from P/Q@CNMPs with the tendency of sustained release stage from 2 h to 12 h and then get a slow release. After 48 h, the cumulative release of the PTX from P/Q@CNMPs reached 43.48% in pH 7.4 PBS while 59.20% and 66.68% were released in pH 6.8 and pH 5.5 PBS, respectively. At the same time, 42.15% of QUE was released in pH 7.4 PBS, while 50.59% and 67.72% were released in pH 6.8 and pH 5.5 PBS, respectively.

### 3.3. Targeting efficiency of P/Q@NMPs and P/Q@CNMPs on NSCLC cancer cells

**Figure 3.**
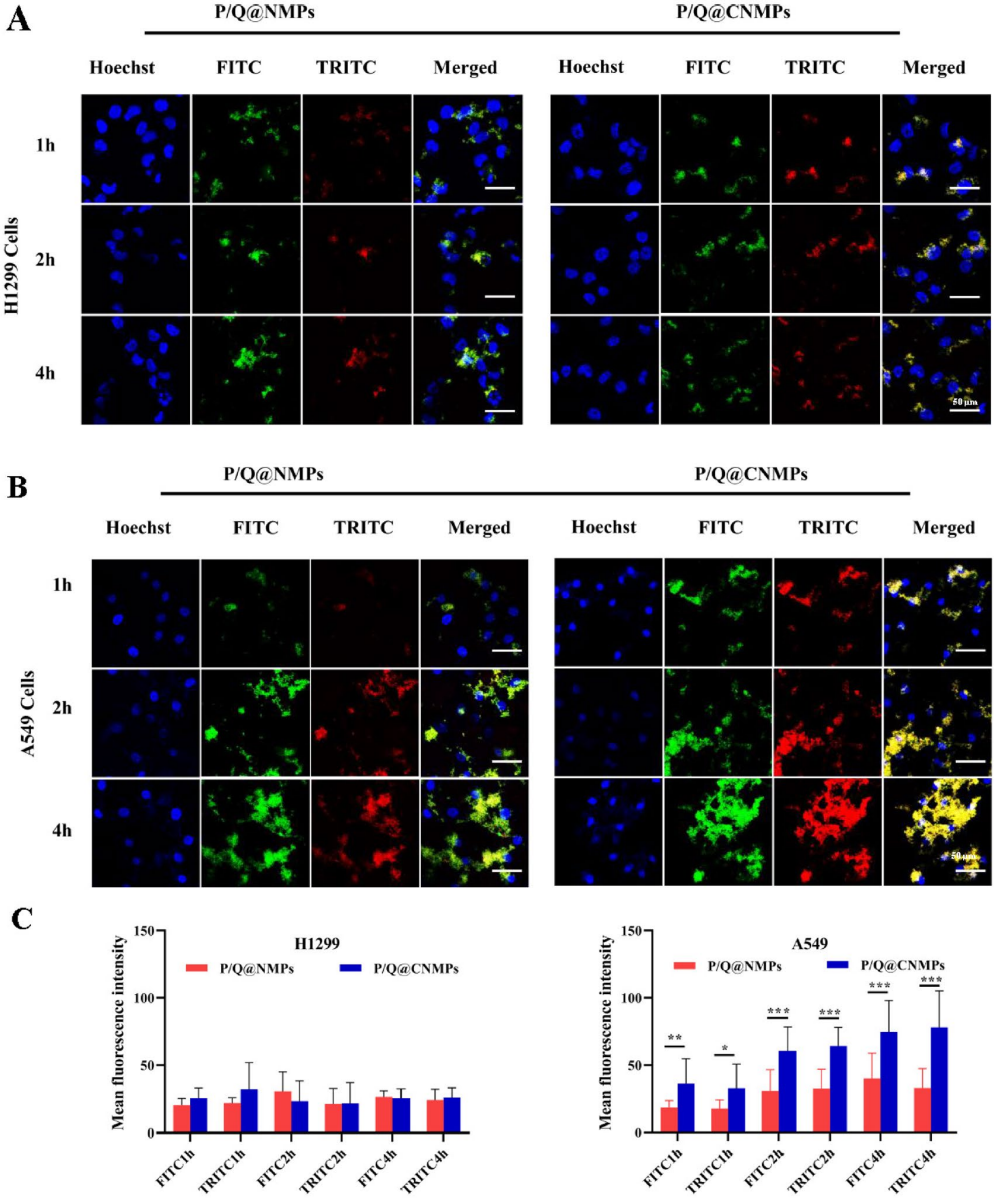
The targeting efficacy of (A) P/Q@NMPs and (B) P/Q@CNMPs in H1299 and A549 cells after co-cultured 1, 2, and 4 h, respectively. The nuclei are stained blue. (C&D) Quantitative histogram of fluorescence intensity. The scale bar is 20 μm. *P < 0.05, **P < 0.01, ***P < 0.0001.

To analyze the targeting efficiency of P/Q@CNMPs *in vitro*, EGFR positive cell line (EGFR^+^) A549 and EGFR negative cell line(EGFR^-^) H1299 cell lines were chosen for this assay. The nucleus was stained with DAPI and P/Q@CNMPs were labeled with both FITC and TRITC which represent PTX and QUE, respectively. As shown in Figure 3(A), there was no significant fluorescence intensity change in H1299 cells with co-incubate time increase, implying that P/Q@CNMPs wouldn’t actively target EGFR low express tumor cells. In Figure 3(B), the results showed that, after 1 h of co-incubation, the P/Q@CNMPs were all present in A549. And as the co-incubation time increased with P/Q@CNMPs, strong fluorescence intensity was observed in A549 cells. Figure 3(C) is the quantitative histogram of fluorescence intensity of H1299 cells and A549 cells treated with P/Q@NMPs and P/Q@CNMPs which meant that P/Q@CNMPs had more cumulative in the A549 cells than in H1299 cells and more target efficiency than P/Q@NMPs. These results supported the analysis of the A549 cells have a nice response to P/Q@CNMPs since the EGFR overexpression in it.

### 3.4. Effect of P/Q@CNMPs on anti-tumor in vitro

**Figure 4.**
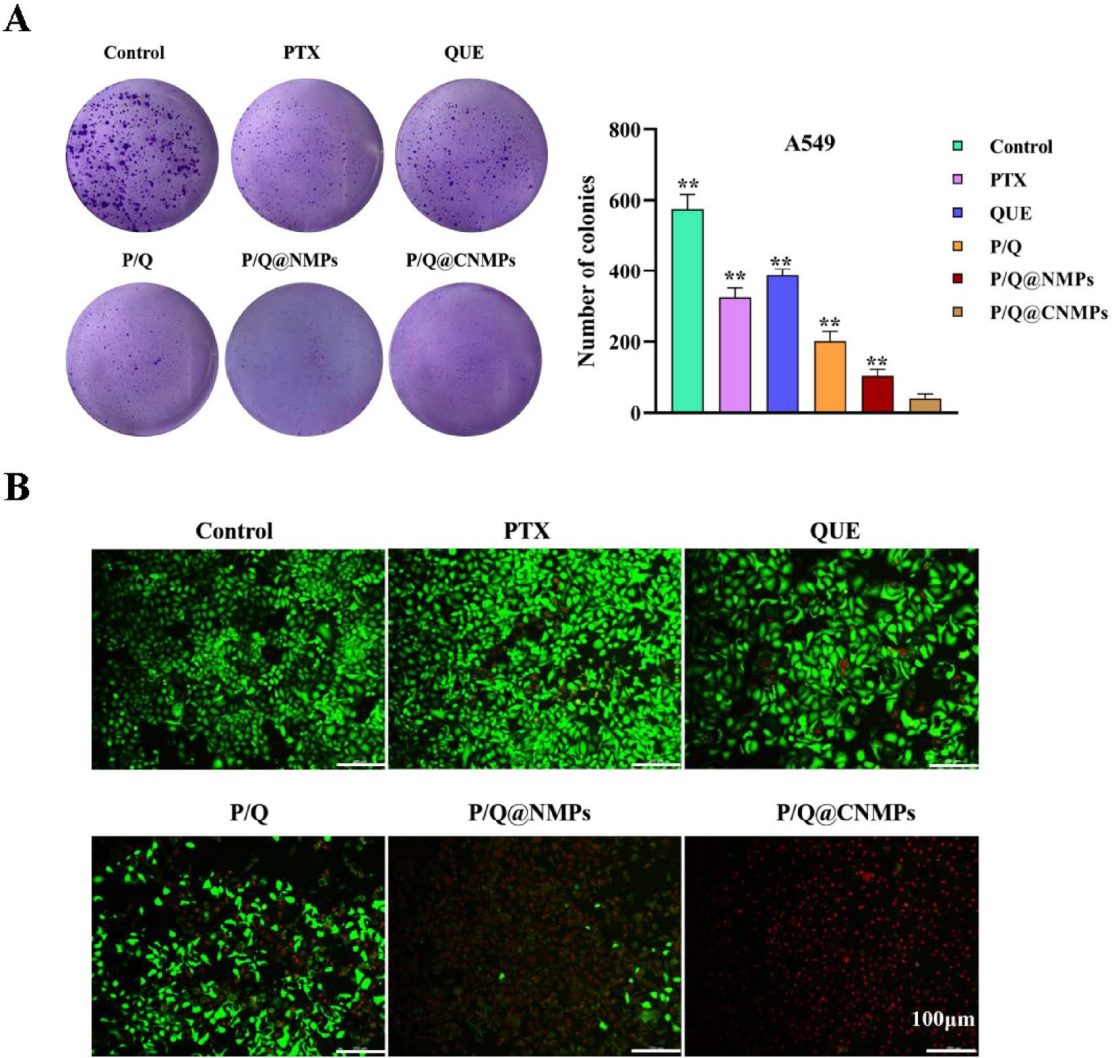
*In vitro* evaluation of anti-tumor efficacy. (A) Colony formation of A549 cells and (B) cell vialibity of A549 cells after treatment with pure PTX, pure QUE, the mixture of PTX and QUE, P/Q@NMPs and P/Q@CNMPs, the scale bar is 100 µm.

The anti-tumor efficiency of the P/Q@CNMPs was observed via colony formation and cell viability assay. As revealed in Figure 4(A), after treatment with different formulations and continue incubation 2 weeks, the colony number of the P/Q@CNMPs group was the least in all the groups and the quantitative histogram of A549 colony formation were consistent with this result. These results presented that P/Q@CNMPs have a strong inhibitory effect on A549 cells.

Cell viability used calcein-AM and propidium iodide kit to observe the status of the cell intuitively. Live cells were stained by calcein-AM with green fluorescence and dead cells were stained by PI with red fluorescence. As observed from Figure 4(B), compared with pure PTX and pure QUE groups, the mixture of PTX and QUE group had higher cell-kill efficiency which suggested that the combination of PTX and QUE have a synergistic therapeutic effect. In addition, compared with other treatment groups, the P/Q@CNMPs group exhibited a much stronger cell-kill effect which was consistent with the cell uptake assay.

### 3.5. Targeting effect of P/Q@CNMPs on NSCLC tumor in vivo

**Figure 5.**
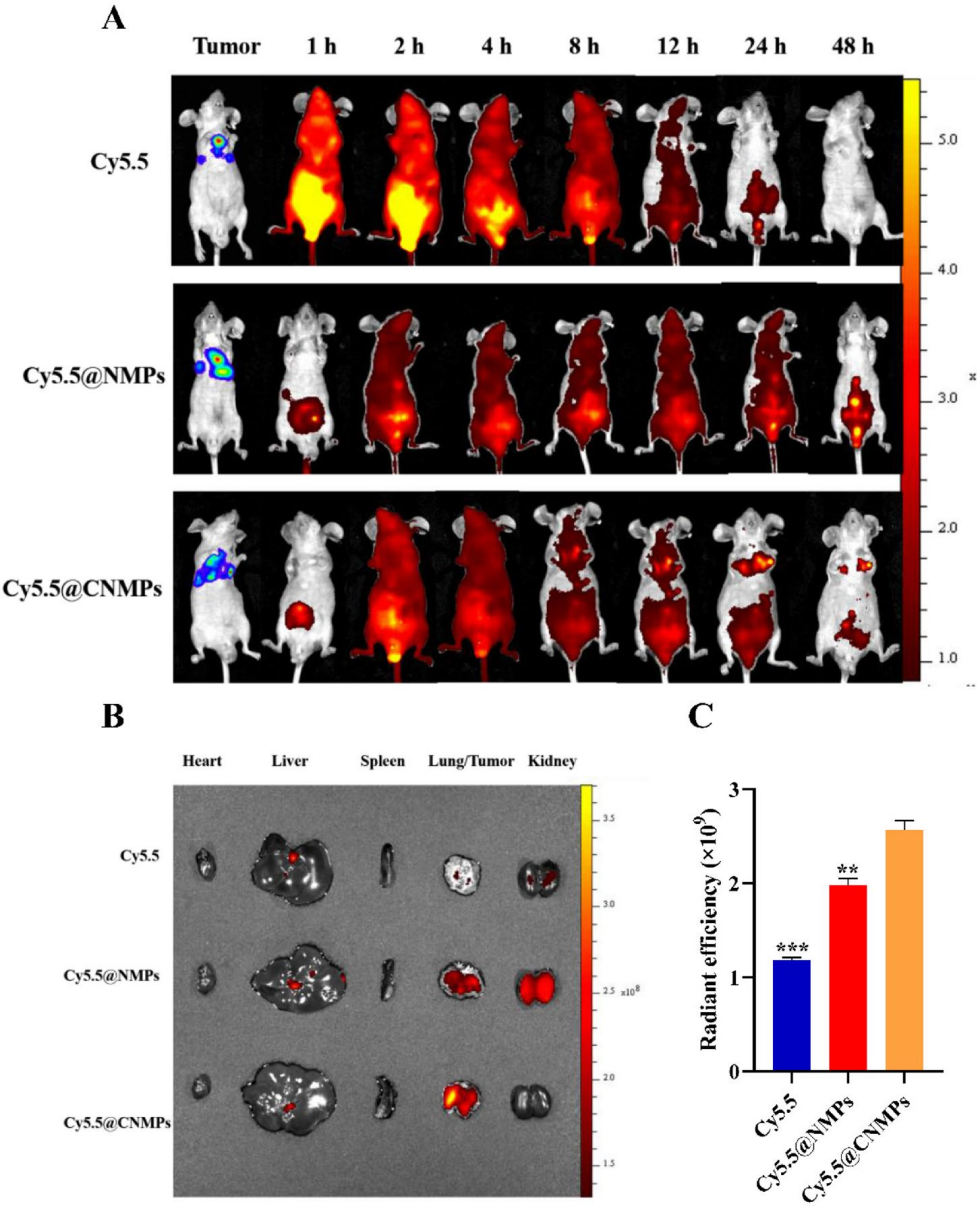
(A) *In vivo* imaging of the orthotopic tumor-bearing mice at different time points; (B) *Ex vivo* fluorescence images of major organs and tumor tissues after injection of pure Cy 5.5, Cy5.5@NMPs, and Cy5.5@CNMPs after 48 h; (C) Quantitative histogram of ex vivo fluorescence intensity. (*n* = 3), **P < 0.01, ***P < 0.0001.

To demonstrate the targeting efficacy of P/Q@CNMPs *in vivo*, the near-infrared fluorophore (NIRF) imaging was used to monitor the distribution status of various formulations *in vivo*. A549 tumor-bearing mice were given pure Cy5.5, Cy5.5@NMPs, and Cy5.5@CNMPs via intraperitoneal injection to be consistent with the tumor treatment. Pure Cy5.5 was rapidly distributed to the whole body of mice within 1 h (Figure 5A). With the extension of time, the fluorescence intensity in mice decreased rapidly and decreased to the weakest after 12 h post-injection. Meanwhile, after the treatment of Cy5.5@NMPs, the cycle time of Cy5.5 fluorescence in mice was significantly prolonged, and there was still fluorescence present *in vivo* after 48 h post-injection. In comparison, after the treatment of Cy5.5@CNMPs, the circulating time of Cy5.5 fluorescence in mice was also prolonged, and strong fluorescence appeared at the site of the tumor from the 4 h post-injection. After 24 h post-injection, the fluorescence intensity at the tumor reached the strongest. After 48 h, the main organs were excised to determine the accumulation of fluorescence intensity (Figure 5B). Figure 5(C) is the quantitative histogram of *ex vivo* fluorescence intensity in tumor, implying that P/Q@CNMPs has the strongest targeting efficiency to orthotopic tumor. These results showed that Cy5.5@CNMPs have more effective accumulation in tumor tissue than Cy5.5@NMPs which was consistent with the result of cellular internalization.

### 3.6. Hematological analysis

**Figure 6.**
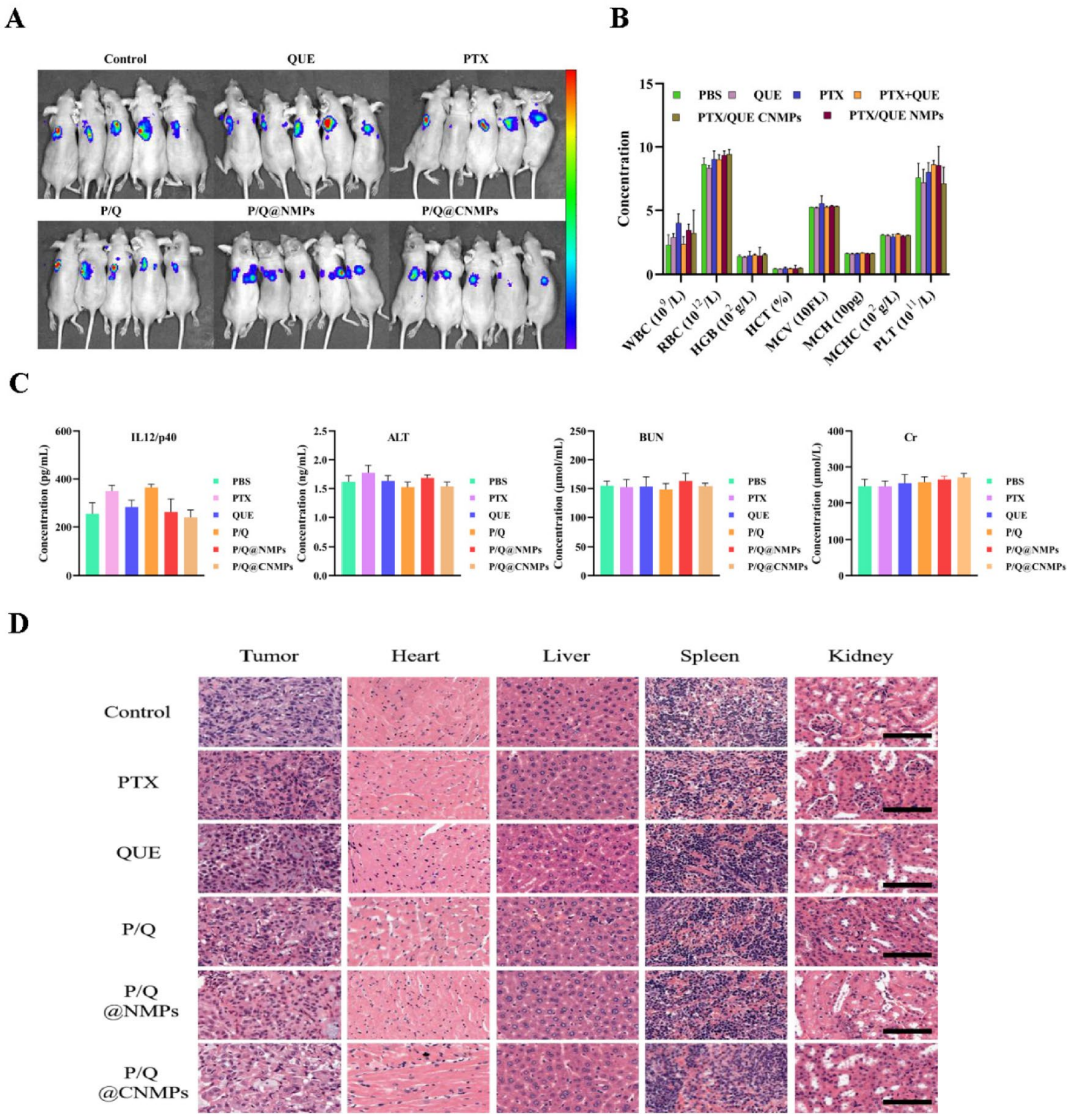
*In vivo* evaluation of anti-tumor efficacy. (A) Representative NIRF photographs of mice tumors after 21-day treatment; (B) Hematological analysis (WBC, RBC, PLT, HGB, HCT, MCV, MCH, and MCHC), (C) blood biochemical analysis (IL 12/p40, ALT, BUN, Cr), and (D) H&E stained histological sections, including tumor, heart, liver, spleen, and kidney, after treatment with PBS, pure PTX, pure QUE, the mixture of PTX and QUE, P/Q@NMPs, and P/Q@CNMPs, respectively. (*n* = 5) Scale bar 100 μm. Abbreviations: WBC, white blood cell; RBC, red blood cell; PLT, platelet; HGB, hemoglobin; HCT, hematocrit; MCV, mean corpuscular volume; MCHC, mean corpuscular hemoglobin.

To confirm the safety of treatment groups in the tumor-bear mice model, hematological analysis was presented in Figure 6(B), there is no significant change between P/Q@CNMPs and the control group. Furthermore, IL12p40 and ALT were the marker of proinflammatory cytokine and acute hepatocyte damage, respectively. BUN and Cr are important indicators of renal function. The result showed that the IL12p40, ALT, BUN, and Cr levels in the P/Q@CNMPs treatment group were at the normal level (Figure 6C). In general, these results suggested that P/Q@CNMPs have low systemic toxicity and good biocompatibility.

### 3.7. P/Q@Cnmps inhibited tumor growth in vivo

To access the inhibited tumor effect of the P/Q@CNMPs, we treated the tumor-bearing mice model with PBS, PTX, QUE, the mixture of PTX and QUE, P/Q@NMPs, and P/Q@CNMPs. As shown in Figure 6(A), the tumor growth inhibition was monitored by NIRF at 21th day of treatment. The intensity of P/Q@CNMPs was the weakest among all the groups. Therefore, the P/Q@CNMPs have a significant inhibition effect on EGFR high-expression NSCLC tumors. As illustrated in Figure 6(D), H&E-stained tissue sections for the histological analysis presented that tumor cells of PBS treatment were disordered and abundant, while with treatment with P/Q@CNMPs the tumor cell intensity was reduced significantly and more cells were dead. The tissue sections for the histological analysis presented that there was no significant damage to the main organs (heart, liver, spleens, kidneys) at the given dosage.

## 4. Discussion

Although chemotherapy is the preferred treatment for NSCLC, its application is limited by drug resistance and toxicity to normal organs. These problems are common and urgent clinical problems that need to seek solutions and overcome. Targeting strategy is an important approach to address these problems because it can increase the concentration of drugs in tumor cells and reduce the toxic side effects on normal tissues (Tekade et al., 2008; Yang et al., 2021). However, there is still a problem of low efficiency in the current targeted system.

Cetuximab which with high binding force to the EGFR receptor expressed on the surface of tumor cells, we proposed it can be modified to the surface of the delivery system and has the ability to drive the delivery system to actively target tumor tissues. In our group’s previous experiments, we occasionally found that the combination of PTX and QUE inhibits resistance to PTX in NSCLC by inhibiting Akt and ERK phosphorylation and MMP depolarization (Wang et al., 2021), based on the fact that QUE could be a chemosensitizer and interferes with the hydrolytic activity of ATPase to alleviate resistance to PTX by noncompetitive binding and blocking efflux. This is a discovery with great potential for the treatment of NSCLC. NSCLC is a lung disease, so pulmonary inhalation is a better way for NSCLC treatment, which can shorten the drug’s delivery distance to the tumor site and improve drug efficacy. Generally, the particle size of inhaled lung particles is in the micron level, 1-5 μm is the easiest to deposit into the lung (Shahin et al., 2013), but to make the particles more easily uptake by cells, we prepared microspheres composed of particles at nano-level (NMPs). When the NMPs enter body fluids, they can be dispersed into nanoparticles which are easy uptake by cells to play the kill effect (Tekade et al., 2008). In this account, Cet was used as a ligand to drive NMPs to specifical target EGFR receptors on the surface of tumor cells, and PTX and QUE were co-loaded by this system as the anti-NSCLC agents (P/Q@CNMPs).

As shown in Table 1, all NMPs’ aerodynamic diameter sizes were range from 2.5-to 4.5 which is generally considered to be the most appropriate size for the inhaler formulation (Stace & Ktistakis, 2006). NMPs also showed positive Zeta potential that can have a stronger binding force with negatively-charged molecules (Okamoto et al., 2019), like lipid phosphatidylserine (Maya et al., 2013), in cancer cell membrane. The high EE rate may be attributed to the spray drying technology could protect the unstable bioactive compounds not degradation from other complex preparation methods. DL rate is an important parameter for the drug delivery system, low DL efficiency means that the drugs loaded wouldn’t easy be affected by the surface destruction of NMPs.

As can be seen in Figure 2(A)–(D), the NMPs were present in a uniform size, and the size ranged from 1-5um, which is assistant with the size detected by the Malvern laser particle size analyzer and the particle size of NPs is about 30 nm which is more suit for cell uptake and could be promoted accumulation by permeability and retention effect (ERP) (Zhang et al., 2011). In Figure 2(E)–(G), the results of *in vitro* release assay showed that in the mimic pH of endo/lysosome (about pH5.5), the cumulative release of PTX and QUE from P/Q@CNMPs was the largest. This phenomenon is major attributed to the process of protonation, the chitosan shell of P/Q@CNMPs will show loose and swollen and present higher solubility, leading to more drugs being released in the medium (Wu et al., 2014). Therefore, we believe that P/Q@CNMPs is a potential control release delivery system for NSCLC treatment.

*In vitro* and *in vivo* targeting assay were shown in Figures 3 and 5, the results showed the P/Q@CNMPs has a higher targeting efficiency than P/Q@NMPs in A549 cells, but in the EGFR negative express cell line H1299, no significant difference between these two formulations. *In vivo* targeting efficiency was detected by NIRF in the orthotopic NSCLC mice model and the result was consistent with which *in vitro* that P/Q@CNMPs could active enrichment into the tumor. These results may be explained by the fact that the ligand Cet had a strong bind force to the EGFR expressed in the surface of A549 and this characteristic of Cet could as the driving force for the delivery system to actively track tumor cells and decrease the side-effects to original cells. The killing effect of P/Q@CNMPs was better than other groups which analyzed by colony formation and live-dead cell detection (Figure 6) which is associated with the higher cellular uptake of P/Q@cnmpS in A549 cells. We also attributed this to the that the P/Q@CNMPs modified with Cet ligand could deliver more anti-tumor drugs (PTX and QUE) to tumor cells than other groups by the EGFR receptor-mediated endocytosis. The flexible combination of mAb and drug delivery carriers enabled us to develop an active target strategy, in which high expression EGFR receptor NSCLC cells will be found in accuracy while the normal cells could avoid injury (Chvatal et al., 2017).

Systematic toxicity and biocompatibility are the very consideration factors for the drug delivery system. In our experiment, we found that P/Q@CNMPs have the low toxicity and good biocompatibility *in vivo* of the treated mice (Figure 6B–D).

The *in vivo* anti-tumor effect was tested for 21 days in the BALB/C-nude mice orthotopic NSCLC model. And the tumor size was shown by bioluminescence; the bioluminescence intensity of the P/Q@CNMPs group was smaller than control and other groups, implying that it has a stronger inhibition effect on A549 tumors (Figure 6A).

## 5. Conclusion

We successfully conducted the Cet-modified nanocomposite microparticles and co-loaded PTX and QUE (P/Q@CNMPs) by spray drying technology. These results demonstrated that P/Q@CNMPs have a proper aerodynamic diameter, high DL capacity, and pH trigger-release ability. *In vitro* and *in vivo*, P/Q@CNMPs have a confirmed inhibiting effect on NSCLC tumors which positive express EGFR. In this account, P/Q@CNMPs have also been approved that it has a good targeting effect on EGFR overexpression of NSCLC cells *in vitro* and *in vivo*. H&E staining and hematological analysis both suggest that P/Q@CNMPs have good biocompatibility and low toxicity to normal tissue. In summary, the P/Q@CNMPs could be a potential for NSCLC treatment strategy through actively targeting tumors.
